# Philanthropy in art: locality, donor retention, and prestige

**DOI:** 10.1038/s41598-023-38815-1

**Published:** 2023-07-27

**Authors:** Louis Michael Shekhtman, Albert-László Barabási

**Affiliations:** 1 Network Science Institute, Northeastern University, Boston, MA 02115 USA; 2 Department of Medicine, Brigham and Women’s Hospital, Harvard Medical School, Boston, MA 02115 USA; 3 Department of Network and Data Science, Central European University, Budapest, 1051 Hungary

**Keywords:** Complex networks, Human behaviour

## Abstract

A significant portion of funding for art comes from foundations, representing a key revenue stream for most art organizations. Little is known, however, about the quantitative patterns that govern art funding, limiting the fundraising efficiency of organizations in need of resources, as well as optimal funding allocation of donors. To address these shortcomings, here we relied on the IRS e-file dataset to identify $36B in grants from 46,643 foundations to 48,766 art recipients between 2010 and 2019, allowing us to quantify donor-recipient relationships in art. We find that philanthropic giving is broadly distributed, following a stable power-law distribution, indicating that some funders give considerably and predictably more than others. Giving is highly localized, with 60% of grants and funds going to recipients in the donor’s state. Furthermore, donors often support multiple local organizations that offer distinct artforms, rather than advancing a particular subarea within art. Donor retention is strong, with nearly 70% of relationships continuing the next year. Finally, we explored the role of institutional prestige in foundation giving, finding that funding does correlate with prestige, with notable exceptions. Our results present the largest and most comprehensive data-driven exploration of giving by foundations to art to date, unveiling multiple insights that could benefit both donors and recipients.

## Introduction

Art and cultural institutions in the US require considerable resources^1–4^. While most institutions operate on limited budgets, a select few raise hundreds of millions of dollars in operating expenses to fulfill their missions. While revenue can come from federal and state governments, and can be earned from sales, for most art organizations philanthropic contributions are the primary source for funding. For example, in 2018 the Metropolitan Museum of Art reported receiving over $250M and the Museum of Fine Arts in Boston reported over $60M in philanthropic support, representing 48% and 45% of their respective total revenue.

Surveys focusing on a few large foundations have offered multiple insights on the drivers of philanthropic giving^2,5^. They showed that the top 1% of museums receive 41% of donated revenues^5^ and noted that the top 1000 foundations gave 9% of their overall grant dollars to art^2^. Other works explored the role of individual donors, and the psychological and individual characteristics that motivate their giving patterns^6–8^.

The emergence of big data and computing power has raised the prospect of significantly expanding upon these works, potentially offering a more granular understanding of the patterns that characterize art funding. For example, the recent availability of the IRS 990 e-file dataset, containing tax returns of all nonprofit organizations submitting electronically, offers researchers an unprecedented level of detail on the activity of both funders and recipients. Here we take advantage of this extensive dataset, allowing us to move our study beyond the largest foundations and explore the full spectrum of donors active in art, including donors whose primary focus is elsewhere and only occasionally support art institutions. Most important, the data helps us reveal the patterns of support characterizing smaller organizations as well, including artist collectives, historical societies, dance groups, and others, representing the vast majority of organizations involved in art.

Our analysis of over 3.6 million tax returns from the IRS e-file dataset helps us offer an unusually comprehensive picture of the patterns that characterize support for art. We show that art funding is highly local, with over 60% of dollars being provided by institutions in the donor’s state. We also find that art institutions within the same geographical area tend to rely on the same funders, largely independent of the artistic experiences they offer, e.g., visual or performing arts. Third, we observe strong donor retention, finding that funders who support institutions for multiple years are increasingly likely to continue their support. Finally, we offer a case study of art museums, finding that funding levels correlate with institutional prestige, with notable exceptions for institutions that engage a unique and captive donor base.

### Prior work

Cultural economics^9^ aims to explain market demand for museums and other art venues^10^, exploring the role of entrance fees^11^ and the diversity of revenue streams that support art^12^. As evidenced by the examples cited in the introduction, philanthropy has been a key, but controversial source of revenue. This controversy about philanthropy is true across all areas of giving^13^, where the impact of private philanthropy on democratic and public institutions has been brought into question. At the same time others have risen to defend philanthropy as a better outlet of wealth than the alternative of continued accumulation in private hands^14^. This debate has been even more acute in art as artists themselves have raised the issue of art funders’ sources wealth and protested against these individuals’ membership on museum boards^15^.

In the academic literature, there has been exploration on whether increased government support leads to decreased philanthropic support, driven by the so-called ‘crowding out’ hypothesis^16–20^. One of the first studies on this topic suggested that the National Endowment for the Arts (NEA) crowded out private donations^19^, though the extent of the effect remains a topic of debate^18,20,21^. Indeed, subsequent work has even suggested the emergence of ‘crowding-in’ effects, where government funding increases private donations^22^. Equally explored is the question of corporate giving, aiming to offer a theoretical understanding of why corporations choose to engage in philanthropy^23–25^.

A key recurring question in philanthropy focuses on the variables that drive donations, both at the foundation level and the individual traits that drive greater giving^6,7,26^. Survey data has explored how particular donors select charities^27^, noting the existence of a gap in understanding specific giving relationships as opposed to studies that have explored aggregated levels of giving based on demographic variables. At a more detailed level, a limited number of works have explored features that increase the likelihood of a donor giving to a specific charity^27^. Geography was shown to be a key factor in donor-recipient relationships, playing a role in several survey studies^28,29^ and various frameworks involving identity and homophily have been used to explain giving^30^. In particular, detailed surveys have shown that donors with stronger ties to the local community tended to donate more^31^ and that athletes are more likely to support their alma mater if they continue to live in the same state^32^.

A second aspect of individual donation relationships is whether the recipient organizations are able to retain their donors thereby attracting sustained revenue to the organization^33^. Sargeant^34^ noted that charities lose around half of their donors who made one-time commitments, and that an additional 30% of donors each year stop donating. Recent work on crowdfunding platforms found even lower levels of retention, with only 26% of donors contributing again one year later^35^. Possible methods to increase donor retention, like more effective communications have also been explored^36^, as well as methods relying on donor identity, commitment, trust, and satisfaction^36,37^.

Finally, within the art sector, status and prestige play an important role in funding. Ostrower carried out a detailed assessment of prestige effects in terms of board trustees, who at the same time support the organization themselves and help fundraise externally on its behalf^38^. Similarly, art institutions confer prestige on artists, driving careers and ultimately artistic success^39,40^, which in turn affects the institution’s ability to attract future funds. Finally, art appreciation and support is often associated with the social class^41–43^.

Despite the tremendous theoretical frameworks and multiple hypotheses tested regarding individual giving relationships, empirical work continues to be limited in scope, typically focused on a few of the largest foundations^5^. For example, when Szántó studied foundation support of art in 2003^44^, he did so by focusing on the top 50 foundations by asset size. Grantmakers in the Arts, a national association of almost 300 public and private funders of art and culture, since 2000 issues an annual report on giving to art^2^, relying again on data from the largest foundations. Work by Osili et al.^45^ explored a larger dataset of nearly 80,000 multi-million dollar gifts collected by scraping public announcements, though only a portion of those grants went to art and this again only focuses on large gifts.

Big data and the accompanying data-driven methodologies have begun to enter philanthropic studies, partly thanks to the opportunities offered by SMU DataArts, Candid and the IRS e-file 990 dataset^46^. For example, researchers have leveraged the IRS 990 e-file dataset to identify organizational activities based on mission statements, enabling research on institutions that share a particular focus or characteristic^47,48^. Another public dataset explored by non-profit researchers captures US federal government awards, where 7% of the grants go to non-profits^49^. Data-based inquiry has also focused on nonprofit board memberships, explored in subsets of regions in the US^50^ and across a swath of foundations in China^51,52^. Recent efforts have also collected data related to social media, measuring how nonprofit advocacy organizations gain following^53^ and how social media is leveraged in fundraising efforts^54^. Lastly, computational approaches were used to map the space of nonprofit research itself, demonstrating the growing interest in the field^55^.

Using Big Data methodologies to explore funding patterns is particularly important for both establishing stylized facts to drive future theory, and for practitioners in considering allocations of resources. Funding of art and culture is well-suited to the application of these methodologies given the increasing availability of large-scale data as opposed to e.g., religion, where many organizations do not have to reveal information to authorities. Likewise, art is a sufficiently large section of non-profit giving and has a distinct categorical nature, enabling the identification of the relevant institutions across multiple levels of support and locations. Finally, a focus on art enables us to compare results to recent work on philanthropic funding in science^56^, and by comparing and contrasting these two major areas of philanthropic support in the US we can separate general patterns from those that are domain specific.

## Data and methods

US-based nonprofit organizations are required to file Form 990, detailing their executive leadership, assets, cash flow, and multiple layers of financial information. These forms are collected and publicly shared by the IRS. Foundations who file Form 990PF, and other nonprofits who file Form 990, report grants given to other nonprofit organizations, individuals, or other entities. The IRS e-file dataset contains the tax forms of all nonprofits who filed electronically since 2013 (note tax forms are typically filed one year later, i.e. forms filed in 2013 contain information about 2012, and some organizations file 2–3 years after the reporting period ends)^57^. We analyzed 3,660,949 forms from 685,397 organizations spanning the entire space of nonprofits including those involved in education, health, human services, environmental issues, religion, and art (see Appendix 1). We next identified 10,338,779 grants listed on the givers’ tax forms and applied a matching algorithm^56^ to determine employer identification numbers (EINs) for 8,186,055 grant recipients (see Appendix 1). Given our focus on art, we next reduced the data to organizations under the category of ‘Arts, Culture, and Humanities’ from the National Taxonomy of Exempt Entities Codes (NTEE), allowing us to identify 149,291 art organizations. It must be noted that while the NTEE classifications have been widely used in philanthropic studies^48^, they remain imperfect and some listed art recipients may be improperly categorized and others might be missing. For example, the Art Institute of Chicago and Pennsylvania Academy of Fine Arts, are classified under education.

**Figure 1:**
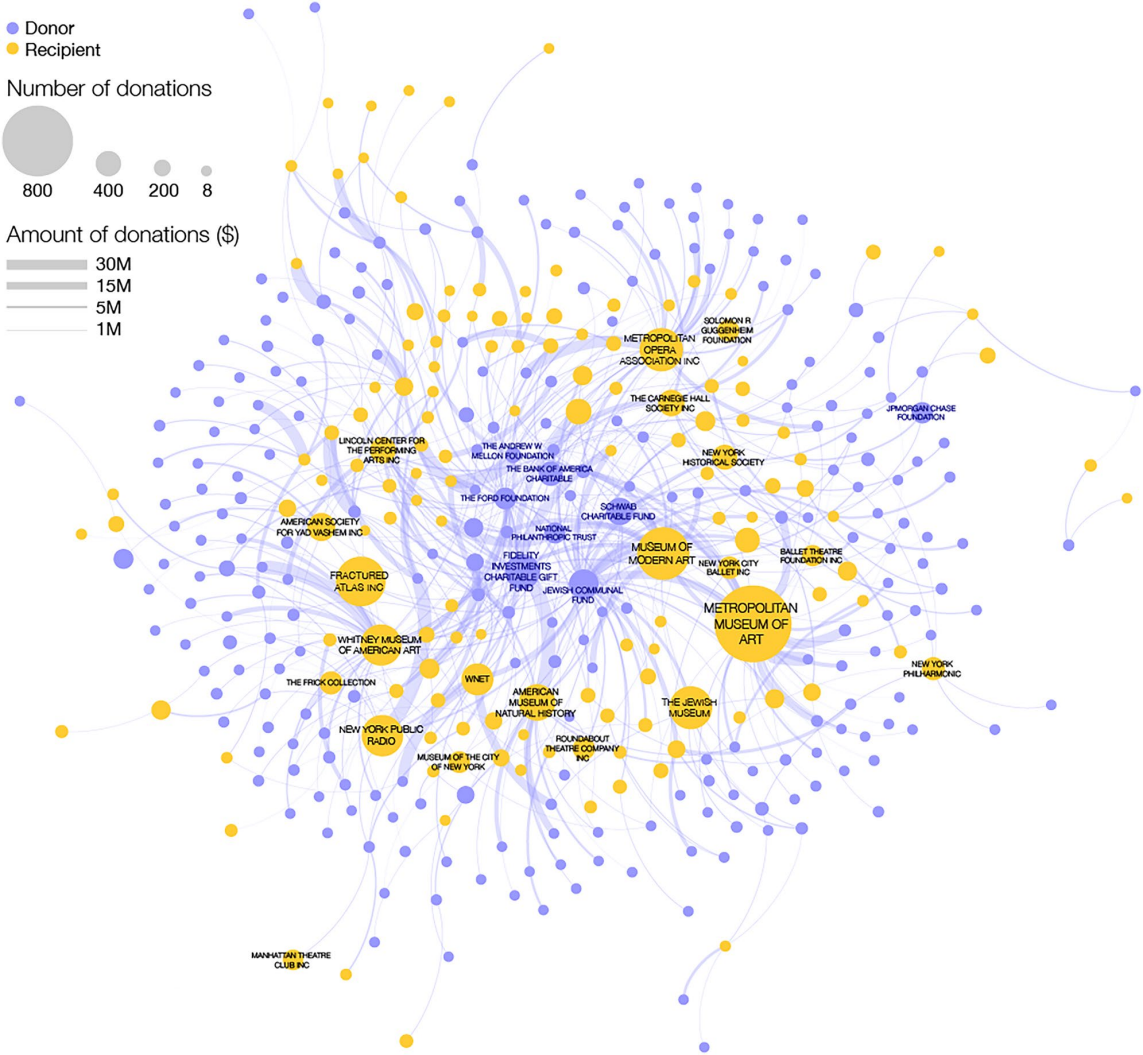
The art philanthropy network. Yellow nodes represent art recipients with a New York City address and purple nodes are donors to those institutions (some of whom may be outside New York). Links show funds given by a donor to a recipient and the width of each link is proportional to the amount of the donation. A core set of donors in the center, including donor advised funds, the Ford Foundation and the Mellon Foundation support multiple art recipients, while other donors in the periphery tend to support a single art recipient. Only donor-recipient relationships involving a total over $1M for the decade are shown.

For each art institution, we mapped grants listed on donors’ tax forms to the institution. To demonstrate the depth of the final dataset, in Fig. 1 we show a subset of the data, capturing grants to art recipients in New York City (NYC). To construct the NYC art funding network, we took each art institution with a NYC address and identified all grants to that institution, arriving at 2144 art institutions who received at least one grant. We then filtered the network to include only grant relationships that totaled over $1M over the course of the decade, leaving 200 recipients and 314 donors. Limiting the network to the largest set of connected donors and recipients, we arrived at 144 recipients and 246 donors connected via 552 grant relationships. The final network shown in Fig. 1 represents the core of New York City art institutions and the major donors supporting them, illustrating the depth of the collected data.

While the data has tremendous richness, it also has known limitations. First, we only have data on foundations who filed electronically and do not have access to paper filers. It is estimated that electronic filers are around 80% of all filers of Forms 990 and 990PF^56^. Second, we relied on a matching procedure to identify the recipient EINs and this matching has accuracy and coverage limitations, identifying only 85% of recipients. Many of the unidentified organizations are foreign groups, public entities such as schools and cities, individuals, and for-profit companies, though some non-profit recipients may also have been unmatched due to filing errors like listing an unofficial name or incorrect address. Among the successfully determined matches, the error rate is estimated to be around 2.6% based on a hand-checked subset of grants^56^. Finally, our data only tracks donations by foundations and lacks information on individual or corporate giving.

### Philanthropic funding in art

**Figure 2:**
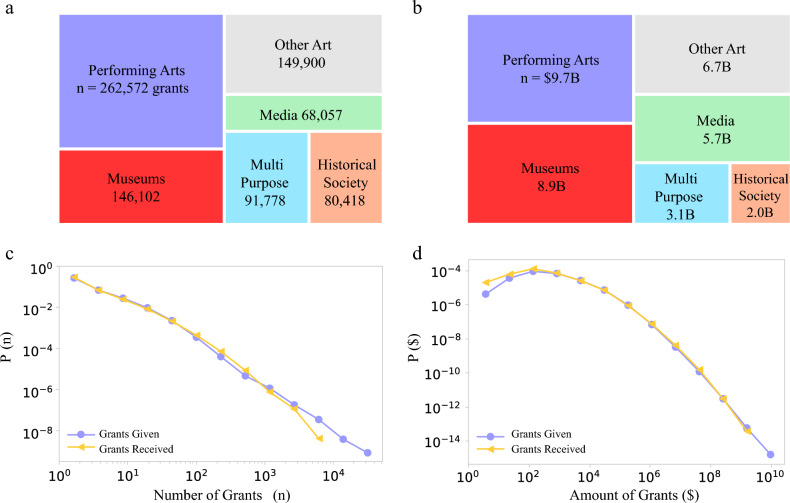
Grants to art and culture. (**a**) The number of grants given to several major areas within art, together representing 798,827 grants. Note that ‘Other Arts’ includes art institutions such as those involved in Art Services, Humanities, and Folk Arts. (**b**) We show the distribution of $36.2B dollars that goes towards art, within the areas of art that received the most funds. (**c**) The distribution of the total number of grants given and received. (**d**) The distribution of the total amount of grants given and received by art organizations. Art funding is focused on museums and performing arts, and other areas receive smaller fractions of both grants and funds. The broad distribution of grants given and received indicates that a small subset of institutions and donors are responsible for a significant portion of funds.

Of the 149,291 organizations categorized under Art, 48,766 (31%) received at least one grant from one of 46,643 donor organizations. Between these organizations, we identified 798,670 grants connecting givers to receivers. The grants totaled $36B over the 2010–2019 decade, reaching $5.9B per year in 2019, considerably higher than the $1.4B that Grantmakers in the Arts, a national association of art funders in the US, estimates to have been given to art in 2018 by public sources, including local, state, and federal entities^2^. Within art, there are multiple types of organizations, as classified by the detailed NTEE codes. Performing arts (NTEE codes beginning with ‘A6’), including, Operas, Dance, Ballet, Theaters, Performing Arts Schools and other subcategories leads both in the number of grants and the largest amount of grants. Indeed the 14,363 performing arts organizations received 262,572 grants totaling $9.7B (Fig. 2). The next largest recipient is Museums (codes beginning with ‘A5’), including art museums, children museums, history museums, and science museums, that together received 146,102 grants, totaling $8.9B. Note that these funds go to only 4463 museums, compared to three times as many performing arts recipients (14,363), implying that on average a museum received $2.0M over the decade, while a performing arts organization received less than half of that, approximately $675k on average. Other recipient areas include 2761 Media organizations ($5.7B, codes beginning with ‘A3’) such as radio, television and film; 7178 Multipurpose Arts Organizations ($3.1B, ‘A2’); 7393 Historical Societies ($2.0B, ‘A8’), and others.

In most subcategories we find more givers than receivers. For example, while there are 393 Opera organizations, the number of organizations that donated to opera is about an order of magnitude larger at 3417. Similar imbalances are present in Ballet, Symphony, and Museums. At the same time, we find that the same group of funders tend to support multiple types of art institutions. For example, over 62% of those who gave to a museum also supported a performing arts institution and 56% of those supporting a historical society also supported a museum. We also find that 20,236 donors gave to a museum, implying that 43% of all funders that gave to art supported at least one museum.

**Figure 3:**
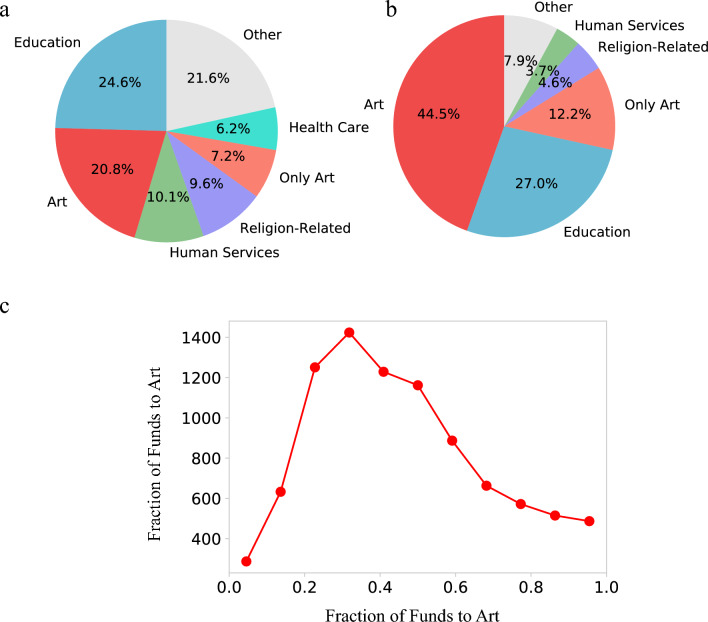
Art is rarely the main focus of art donors. (**a**) The primary interests of art donors as measured by where they gave the most funds. Only 28.0% of art donors gave most or all their money to art. Many art donors have a primary focus in Education, Human Services, Religion, or Health Care. (**b**) Donors with a primary focus in art make up a large fraction of the total funding to art (56.7%), though nearly half of art funding comes from donors whose primary focus is in other areas. (**c**) For donors whose primary focus is art, we show the fraction of their funds that went to art. Even for donors whose primary focus is in art, only 20–60% of their total giving goes to art.

Beyond competing with other art institutions, art recipients also compete for donor funds with other areas as well. Indeed, only 28% of art donors gave either a plurality or all of their grant funds to art institutions (Fig. 3a). In contrast, close to half of the funds (43%) came from donors whose main area was outside of art (Fig. 3b), suggesting that art institutions tend to rely on donors whose primary focus is not art. Finally, even those with a primary focus on art often only gave 20–50% of their funds to art (Fig. 3c).

To place these results in context, we compare the patterns characterizing art funding to those patterns observed for science philanthropy. While there are four times more art organizations than science organizations (34,091 science organizations versus 149,291 in art), 39% of science organizations received at least one grant, compared to 31% of art organizations. The biggest difference is in the total amount of funds given to science from 2010 to 2019, which was $207B, nearly 7 times the amount given to art. Furthermore, science benefits from substantial government funding, including $40B/year from the National Institute of Health and nearly $10B/year from the National Science Foundation, both part of a total $65 billion annual US federal science budget^58^.

It has been previously suggested that the comparatively fewer organizations carrying out science as opposed to funding science is due to the fact that science often requires high costs for equipment and implementation^56^. Art on the other hand can be less capital intensive, leading to a greater balance between the number of donors and recipients, though certain areas of art, like museums, do require considerable funds to maintain their activity.

Taken together, we find that in art there are more organizations competing for less funds from fewer funders compared to science (see Table 1). At the same time, it is important to clarify that science organizations, like universities, tend to contain multiple independent departments and research labs, each competing independently for funds, thus the competition in science is greater than it might appear based on the number of recipients.

**Table 1:** Philanthropic support of art, compared to philanthropic support in science^56^.

	Art	Science
Number of Givers	46,643	58,524
Number of Recipients	48,766	13,531
Number of grants	798,827	926,081
Total Amount of Grants	$36B	$208B

Comparing art to science shows that science has more donors and fewer recipients, and science institutions received more grants and nearly six times more funding over the decade from 2010 to 2019.

Aside from the larger number of recipients, art is also distinct from science in that the primary focus of art funders is often outside of art. Indeed, less than 60% of funds came from organizations whose primary focus is art (Fig. 3b), in contrast over 90% of funds donated to science comes from funders whose primary focus is science (i.e., gave more to science than to any other category).

### Locality in art

**Figure 4:**
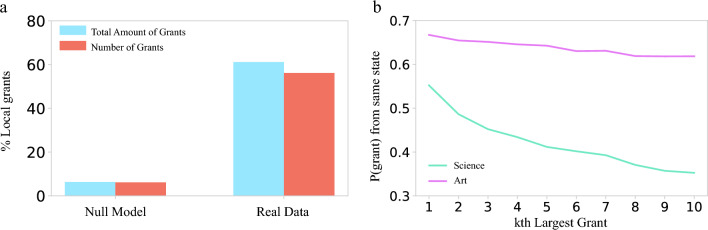
Art funding is local. (**a**) The number and amount of grants given in the same state compared to a national null model indicates that the fractions of dollars and grants given locally is significantly greater than random. (**b**) The fraction of donors whose *k*th largest grant is given in the same state as the donor’s location. While science donors’ 10th largest grant is local only 35% of the time, in art this number is 61%, meaning that an art donors’ smaller grants are local at a considerably higher rate.

Funders’ frequently stated preference for supporting their local communities^31^ raises the question: To what degree is philanthropic funding local, and what role do global funders with national reach play in art? We measure locality by comparing the reported recipient state to the donor’s state of incorporation. We find that 61% of dollars and 56% of grants are local, meaning that the recipient is in the same state as the giver (Fig. 4a). Both numbers are significantly greater than expected if donors dispersed funds nationally, disregarding local interests. Indeed, a degree-preserving national null-model (see Appendix 2), which maintains the number of grants given and received for each funder and recipient, but randomly shuffles the recipients nationally, predicts that less than 5% of dollars and grants would be local.

We find that 22,223 funders (48% of all funders) support exclusively local art institutions (same state as the funder). Just over half of these (11,633) only gave to a single art recipient, while 899 gave to at least 10 different local art recipients and 26 gave to 50 or more art recipients in their home state. Some of these 26 major local funders are local art or humanities organizations with a stated local mandate. There are, however, many private foundations with an exclusive local focus, such as the Char and Chuck Fowler Foundation in Ohio that gave to 81 Ohio-based art organizations, the Reser Family Foundation that gave to 69 different Oregon-based recipients, and the Lily Auchincloss Foundation that gave to 102 art organizations in New York. Interestingly, major national foundations that support art across the US tend to nevertheless have a local emphasis. For example, 47% of the 490 art organizations receiving support from the Macarthur Foundation are in Illinois, 49% of the 660 art organizations receiving from the Ford Foundation are in New York, 41% of the 353 organizations receiving from the Mellon Foundation are in New York, and 54% of the 229 organizations receiving from the Getty Trust are in California.

The fraction of dollars distributed locally is similar to the fraction observed in science philanthropy, however we find that the 56% of grants awarded locally in art is considerably higher than in science, where only 35% of grants were local. This suggests that while in science smaller grants may be given to more distant institutions, in art, even smaller grants tend to be local. Indeed, in Fig. 4b we see that art funders’ 10th largest grant is still local in over 60% of cases, in contrast with science where the 10th largest grant was local in only 35% of cases. Furthermore, in science the largest grant was local in 50% of cases, indicating that science funders’ largest grants are more likely to be local than their smaller ones, while in art this ratio is roughly constant.

The observed differences between science and art are partly rooted in the experiential aspects of art. While one can visit a scientific lab, most science funders rarely do so, since personal experience and engagement is not the primary aim of science philanthropy. In contrast, for most forms of art, access to special events and preferential donor treatment, can be an important motivation for giving^43^. Thus, whereas in science a donor may choose to support research at a distant university if the focus of the work advances the donor’s personal and philanthropic goals, an art donor is more likely to support local institutions whose exhibitions or performances are readily accessible to both the donor and their community.

**Figure 5:**
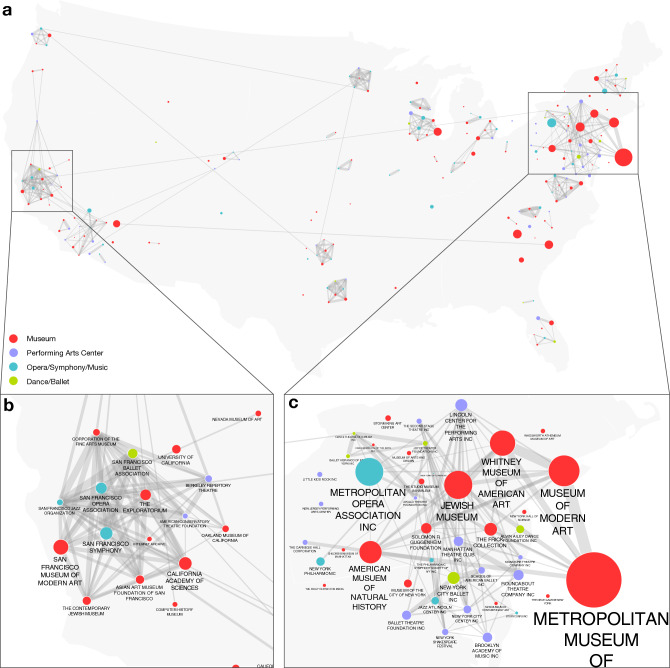
Donor overlap in art. (**a**) Nodes represent institutions and links represent shared donors. We limit the nodes to organizations receiving funds from at least 50 distinct donors and a total of $10M over the decade. Link widths reflect the Jaccard of the set of donors to each institution. Only links with Jaccard weight above 0.1 are shown. Institutions in the same city tend to have high donor overlap even when offering distinct experiences. At the same time, there are very few links across the nation, indicating that institutions with similar artistic offerings in different geographic regions rarely share donors. We show closeups on the regions of (**b**) San Francisco and (**c**) New York.

**Figure 6:**
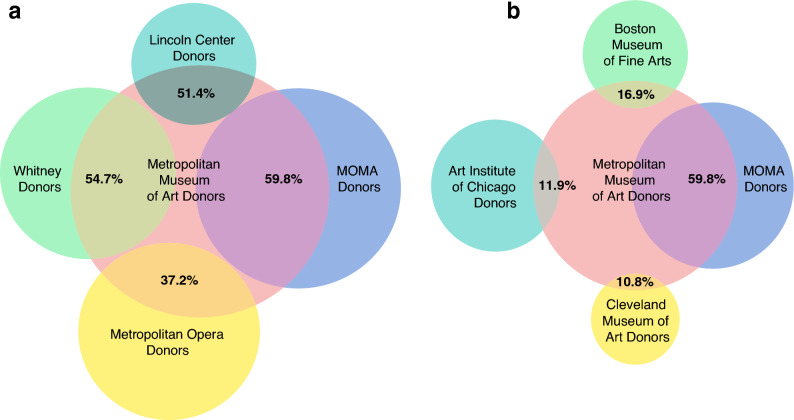
Funder overlap is local, rarely global. For different institutions, we show what fraction of their funders also gave to the Metropolitan Museum of Art. (**a**) Institutions in New York, even if they offer seemingly distinct experiences from the MET, have high rates of overlap in their funders. (**b**) Art museums outside of New York tend to have a low fraction of their donors overlapping with the Met, indicating that art funders give to a variety of local institutions rather than distributing nationally among top institutions offering similar experiences. For additional examples see Appendix Figs. A1, A2.

An important consequence of this local focus of funders is that art organizations from the same local area tend to rely on the same funders even if they offer different experiences, or produce distinct art forms. To show this we built a network of recipient institutions who are connected if they receive funds from the same donors (Fig. 5). We see that within each major metropolitan area of the US there are strong cofunding patterns, even when the recipient institutions offer distinct experiences. For example, in New York, the Metropolitan Museum of Art (MET) received funds from 1374 donors, more than any other art institution. Many donors of other major New York City-based institutions also tended to donate to the MET, including 60% of MOMA’s donors, 55% of Whitney donors, 51% of Lincoln Center donors, and 37% of the Metropolitan Opera’s donors (see Fig. 6a). For example, the Leon Levy Foundation reported 2019 grants of $228,988 to MOMA, $162,000 to the Metropolitan Museum of Art, and $10,000 to the Lincoln Center. In contrast, geographically distant institutions, even if they offer similar experiences, rarely or never share many donors (Fig. 6b). For example, the Art Institute of Chicago shares only 10% of its donors with MOMA in New York and only 12% of its donors with the MET, despite having overlapping artistic profiles and missions. This confirms that competition for funds in art is focused on the region rather than the topic.

To a large extent, the local focus in art funding that we documented above is expected as art funders are often explicit about building local capacity in art to benefit their communities. At the same time, the degree of locality in funding of art is surprising given the trends towards globalization in many areas over recent decades. Moreover, such local funding raises issues of fairness and equity: the source of wealth of most large foundations is rooted in national or international enterprises. The redistribution of this wealth to local institutions can exacerbate existing inequalities, as most foundations are based in already wealthy areas of the US.

### Stability and donor retention in art

**Figure 7:**
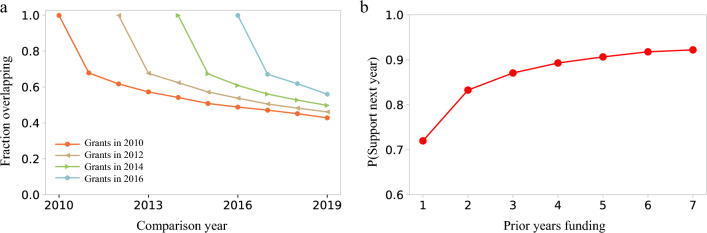
Philanthropic grants are stable in time. (**a**) The fraction of grants overlapping from one year to the next. Only grantors whose tax forms are available in both the original year and the comparison year are included. (**b**) The persistence of grants over time as represented by the likelihood of a grantor giving a grant next year based on the number of previous years they gave.

An important factor in philanthropic funding is donor retention, capturing the degree of recurrent support over multiple years^35,36^. In art, receipt of a prior grant is a strong predictor of future relationships between the donor and recipient. Indeed, we find that 68% of grants repeat one year later, 57% are continued three years later and 50% last 5 years or more (Fig. 7a). These numbers far exceed donor retention for a crowdfunding platform, where only 26% of donors gave one year later^35^. For foundations, retention strengthens over time, as those who gave 2 years consecutively gave again in 80% of cases and those who gave 7 years straight continued to give the next year in 90% of cases (Fig. 7b). We also find that donors who give fewer grants to art organizations are more likely to consistently support the same recipient, with almost 70% of donors with only a single recipient supporting that recipient for 7 consecutive years from 2013 to 2019. For donors giving to 10 different recipients, only around 30% of their recipients received support every year from 2013 to 2019 (Appendix Fig. A3). Similarly, grantors who gave more funds over the decade were more likely to have given annually- those who gave only $1000, gave annually in just 20% of cases, whereas those who donated over $1,000,000, gave annually in 60% of cases (Appendix Fig. A3). The strong donor retention in art can enable institutions to develop financial plans based on likely future revenue from contributions. At the same time, donor retention tends to benefit established organizations and the funds earmarked over many years represent barriers for funding new organizations or programs.

### The role of prestige in art funding

Prestige captures the community’s perception of an organization’s influence and role, and likely plays an important part in an institution’s ability to attract funding. To explore the role of prestige, we rely on the quantitative approach developed by Fraiberger et al.^40^, which mapped the movement of artists between institutions, building a network of institutions linked by artists they both exhibited. Centrality in this network was found to strongly correlate both with the perceived prestige of an institution as coded by experts, as well as the market value of the exhibiting artists. We use the prestige values from Fraiberger et al., to examine the effect of prestige on an institution’s ability to attract funding. We limit the data to US museums and match these institutions to those included in the funding dataset, arriving to 609 US museums and non-profit galleries for which we have both prestige scores and funding profiles (see Appendix 1).

**Figure 8:**
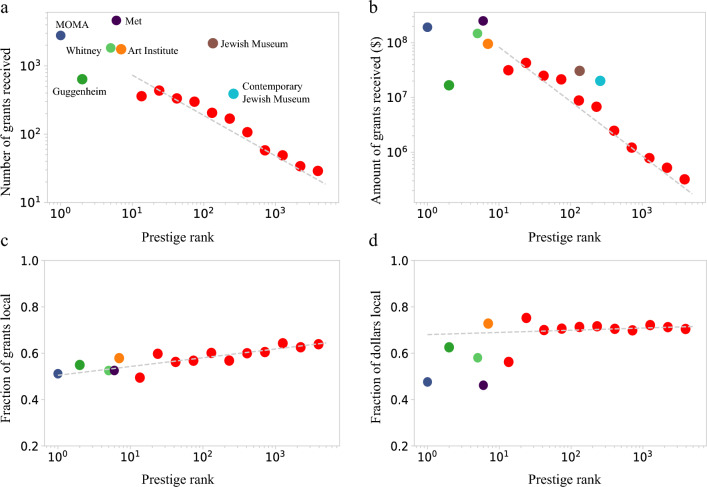
Institutional prestige affects philanthropic support. The number (**a**) and amount (**b**) of grants received by an institution (museum/gallery) as a function of the institutional prestige. The decreasing trend indicates that higher prestige museums receive greater philanthropic support. (**c**) The fraction of grants from local sources increases with decreasing museum prestige. (**d**) The fraction of grant dollars that come from local sources is largely independent of museum prestige.

We find a strong correlation between prestige and the number of grants (Pearson Coefficient = − 0.27, Spearman Coefficient = − 0.49, p < 1e−10) and the total dollar amount of grants received by an institution (Pearson Coefficient = − 0.25, Spearman Coefficient = − 0.51, p < 1e−10). Indeed, the ten most prestigious institutions (top 1.7% of matched organizations) received over 1000 grants each on average, while lower-prestige institutions received from dozens to a few hundred grants (Fig. 8a). We also observe anomalies: the second-highest prestige institution, the Guggenheim Museum, receives less philanthropic support than institutions of similar prestige, likely because of its square footage, limiting it to a single exhibition at any time, compared to its high prestige peers that have multiple exhibition venues. Another outlier at rank 134 is the Jewish Museum in New York with 2146 grants, whose generous support is partly explained by its unique focus. In terms of the amount of grants (Fig. 8b), we observe a similar trend, with the most prestigious institutions attracting over $100M USD over the past decade, while lower-prestige institutions received in the range from $100k to $10M. An outlier at rank 260 is the Contemporary Jewish Museum in California which received over $20M dollars.

We find that less prestigious institutions receive a greater fraction of their funds from local donors (Pearson Coefficient = 0.09, p = 0.03; Spearman Coefficient = 0.119, p = 0.004) though in terms of magnitude this increase is small (Fig. 8c). In terms of the amount of grants (Fig. 8d), the top 10 most prestigious institutions tend to receive a somewhat lower fraction of their philanthropic funding from local sources, though the trend is not significant (Pearson Coefficient = 0.01, p = 0.85; Spearman Coefficient = 0.07, p = 0.12). Overall, this suggests that more prestigious institutions might have greater success attracting donors outside of their local region than less prestigious institutions, though establishing the existence and the extent of this effect requires further data and research.

Disentangling the causal relation between prestige and funding, i.e., is a museum prestigious because it has access to funding or it receives funding because it’s prestigious, is challenging. The founding of new art museums, such as The Broad and Neue Galerie, supported by wealthy philanthropists may provide insight on this matter. As society and the art world’s focus on issues of equity and diversity has increased lately, it will be interesting to see how the renewed focus on diversity could alter both funding and notions of prestige. Will the most prestigious institutions diversify their offerings and continue to attract the bulk of funds or will institutions who currently have less prestige, but greater diversity, manage to attract more funds?

## Discussion

Our data collection and modelling efforts offer a particularly detailed quantitative picture of art funding. Whereas prior research was limited to a few large donors, here we capture giving by all nonprofit organizations, enabling a complete, multi-scale snapshot of funding in art. The patterns we uncover indicate that art philanthropy is locally focused, with high donor retention, and a strong emphasis on institutional prestige. While some of these patterns have been seen in previous studies^27,38^, quantification and broad empirical confirmation has been lacking.

Our findings have multiple implications. For institutions seeking funding, our results highlight the challenges they face and indicate that the competition comes from multiple areas, both from peer institutions as well as institutions not involved in art. At the same time, this finding suggests that reaching out to major donors in their region who have not previously donated to art might lead to success as donors whose philanthropic focus is in other areas are often open to giving a portion of their funds to art. Within art, competition is fundamentally at the local level, often with organizations that offer entirely different artistic experiences. This overlap in donors means that recipients may lose out on potential funds to nearby recipients, but it also means that they have good chances of obtaining funding from donors that already gave to other local art organizations. Attempts by art institutions to identify and solicit other institutions’ donors could lead to donors overall giving a higher fraction of their assets to art.

While it is accepted that donors have a right to allocate their funds, it seems fair to ask if the processes they use are optimal. As philanthropy moves towards more objective measures of impact, it will be interesting to see if data-driven empirical efforts also extend to the art world and if they affect some of the current allocation practices regarding locality, donor retention, and prestige. Indeed, millennials have been found to have a greater desire for measurable impact^59^, which could shift their philanthropic focus to advancing particular areas of art as opposed to funding particular geographic regions. Identifying such shifts in a timely manner will require not only continued efforts to monitor foundation grants as reported in IRS 990 tax forms, but also increased tracking of major individual donors, such as those listed in art organizations’ annual reports. Collecting and analyzing these and other big datasets will provide unprecedented insight into funding patterns in art and could improve funding allocation for the public good.

## Supplementary Information

Supplementary Information.

## Data Availability

To support further work, we have posted the network dataset and code used here at https://osf.io/m7qn9/?view_only=6f078def3b2a4e42874a42c62c009caa to enable other researchers to analyze grants in art.
