# Supplementary material for: Philanthropy in art: locality, donor retention, and prestige
Source: Sci Rep. 2023 Jul 27;13:12157. doi: 10.1038/s41598-023-38815-1 (PMC10374628; doi:10.1038/s41598-023-38815-1)
Supplement: Supplementary file 1 — Supplementary Information. [file 41598_2023_38815_MOESM1_ESM.pdf]

Appendix 1: Addition Data Collection and Processing Information

Our initial data collection and processing follows prior work that analyzed philanthropic grants to science (Authors, 2022). Below we review procedures described there and detail some of the unique data cleaning steps for art.

Initial Data. To track philanthropic grants, we collected the IRS 990 filings from https://registry.opendata.aws/irs990/ in 2021. It is worth noting that the IRS 990 dataset is hosted by the IRS itself since the beginning of 2022 at https://www.irs.gov/charities-non-profits/form-990-series-downloads. The dataset we collected consisted of 3,910,398 tax forms for 685,397 organizations, with 3,660,949 filings for filing years from 2010-2019. We note that 2,281,346 (62%) of the included tax forms had a filing period ending Dec. 31st, though 1,379,603 (38%) filings did not use the standard calendar year for reporting and the year of the filing period end date was used to assign grants from these organizations to a particular year.

Disambiguating Organizations. We focused on the over 10 million grants disclosed on donors' tax forms. Those filing IRS form 990 represent 3,678,608 grants (35%) and must list the recipient's Employer ID Number (EIN), uniquely identifying the grant recipient. The remaining 6,710,171 grants come from returns of private foundations who file Form 990PF, which only requires a name and address of the recipient to be provided. We then worked to identify the EINs of 990PF filers. We first excluded 224,949 grants made to recipients outside of the US. We then used the IRS business master files (BMF) to match organizations by name and state of incorporation. Specifically, we used sklearn Python package's TFIDFVectorizer with word-based substrings and found possible name matches in the same state as listed on the donor tax form. We assign a preliminary match based on the possible match with the maximum cosine similarity. Matches that either have a cosine similarity above 0.75 or have a

cosine similarity above 0.5 and also a zip code that is identical to the zip code listed by the foundation are preserved.

Note that due to disambiguation issues, we removed the Harvard University Band, an art organization, because the band was listed as receiving far more funds than it reported in its own tax forms, presumably because some grants to Harvard University were misassigned by our matching algorithm as going to the band.

Benchmarking Foundation Grants. We note that the dataset we explore includes only giving by foundations and not direct giving by individuals or corporations. When examining a subset of 13,230 art recipients in 2018 who also reported revenue from contributions on their tax forms, we find that they listed total contributions of $23B of which only $4.9B (21%) correspond to grants from foundations. While representing a minority of overall contributions, foundation giving still represents a critical part of operating expenses and likely shares many features with direct-giving by individuals.

Matching to Fraiberger et al. (Fraiberger et al., 2018) dataset. We matched our dataset to nonprofit US listed museums and galleries in the Fraiberger et al. dataset. The data we attempted to match included 1191 museums and 3507 galleries based in the US. We successfully identified 497 museums and 112 galleries, which were handchecked to confirm appropriate matches. We also handchecked the remaining museums to make sure that we did not miss any matches. Many of the missing museums are ones affiliated with universities or larger umbrella groups like the Smithsonian and therefore are not distinct legal entities, precluding the disambiguation of grants to them from those to the parent entity.

Open Data To support further work, we have posted the network dataset and code used here at https://osf.io/m7qn9/?view_only=6f078def3b2a4e42874a42c62c009caa to enable other researchers to analyze grants in art.

Appendix 2: Degree-Preserving Network Null Model Description

To compare local giving while controlling for the fact that there are naturally more institutions in different locales, we use the degree-preserving null model (Newman, Strogatz, & Watts, 2001). The null model preserves the number of grants both given and received (degree) by each individual organization, yet shuffles the recipients of those grants, such that an organization that in the real data gave 10 grants will in each null model realization also give 10 grants and similarly for the receivers. The fraction of grants and dollars given locally in each of 20 random degree-preserving realizations is measured and the average is taken to compare with the real data.

[Figure A1 Here]

[Figure A2 Here]

[Figure A3 Here]

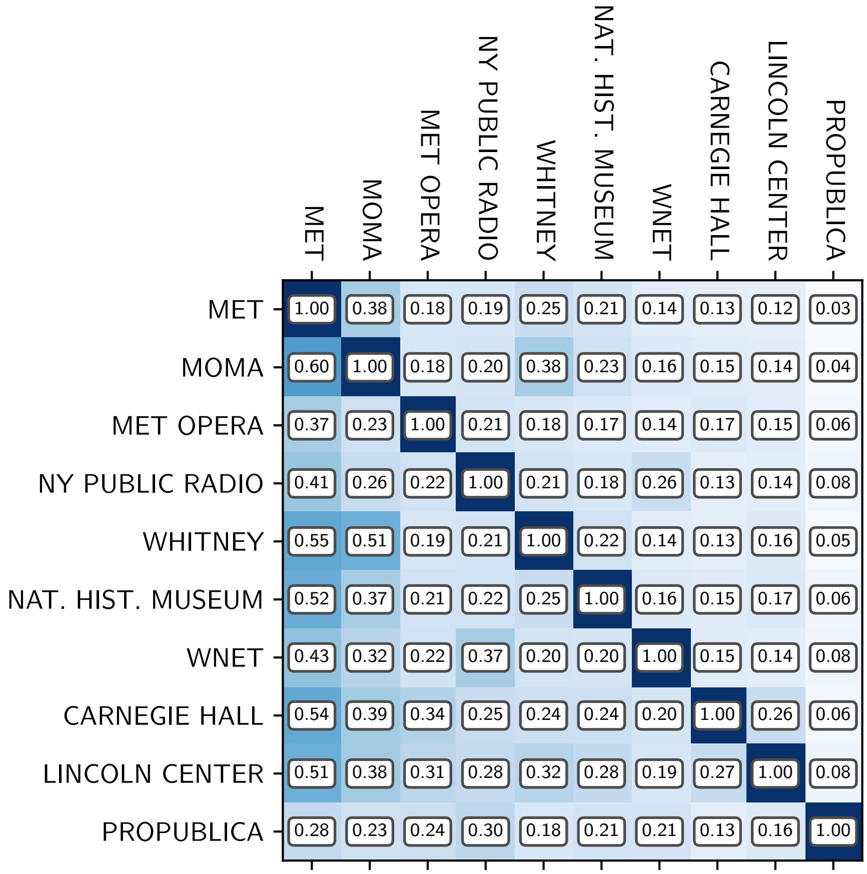

Fig. A1. Local Funder Overlap. We show the fraction of funders to one institution who also gave to another institution in New York. We find that even institutions offering seemingly distinct experiences tend to have high rates of overlap in their funders, demonstrating that art funders give to a variety of local institutions rather than distributing nationally among top institutions offering similar experiences.

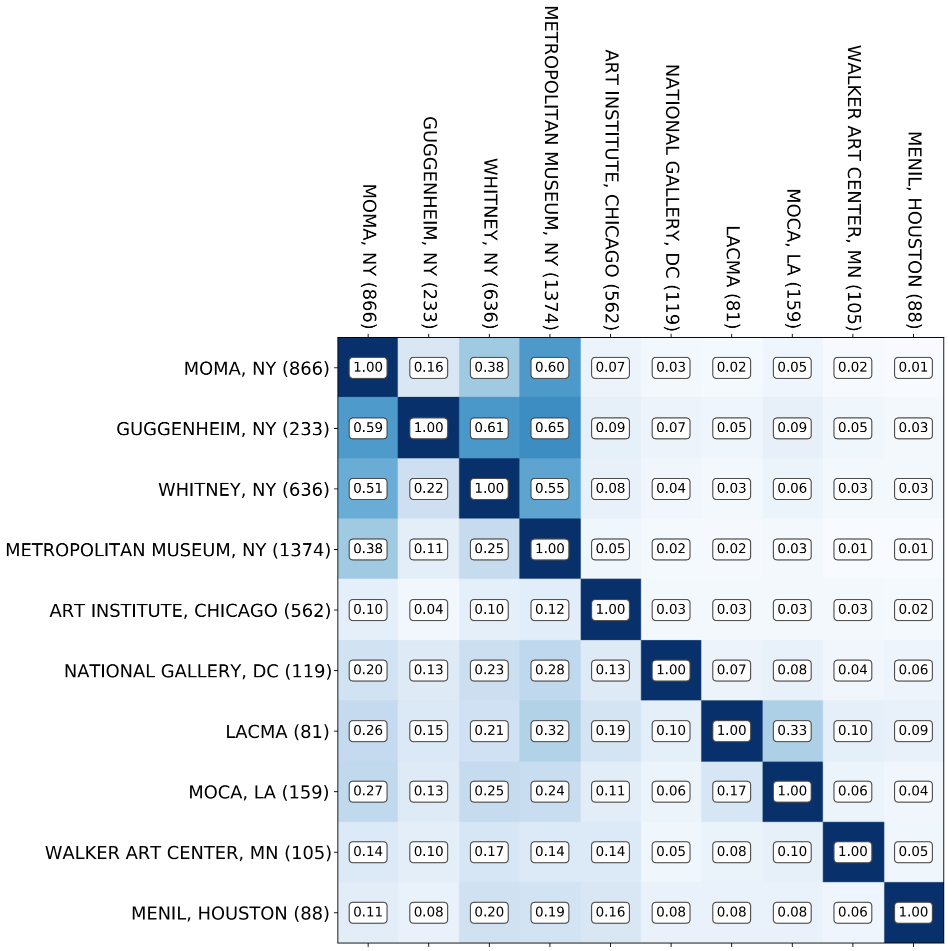

Fig. A2. Museum Funder Overlap. We show the fraction of funders to one art museum who also gave to another top art museum. Next to each art museum is the number of foundation donors to that museum. Distant art museums tend to have distinct donor pools despite offering similar experiences.

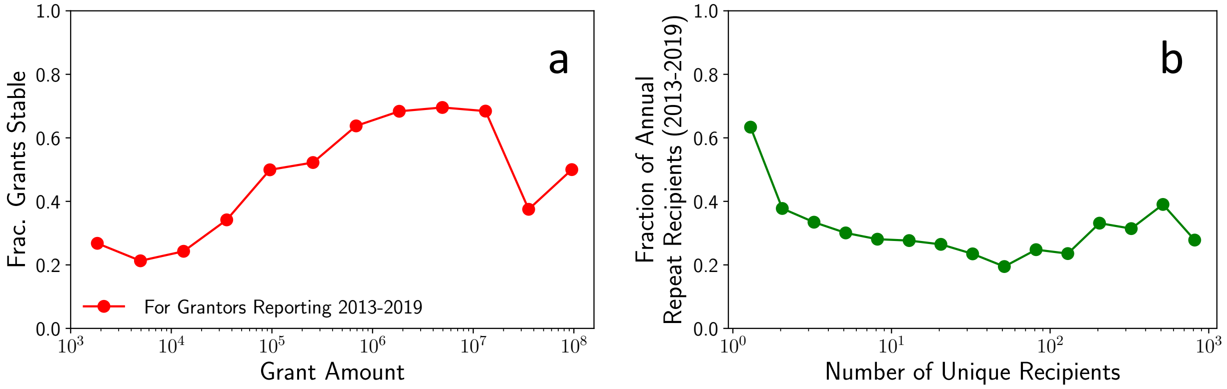

Fig. A3. Annual Grant Giving. (**a**) For donors whose total support for art was at different levels, we see that those who gave only $1,000-$10,000 per year gave to the same recipient annually from 2013-2019 in only around 20-30% of cases. However, those who gave a total of $1M gave to recipients annually in over 60% of cases. (**b**) Donors with only a single recipient gave to the same recipient annually from 2013-2019 in over 60% of cases whereas those with more recipients were less likely to give annually.
